# When Intuition Meets the Algorithm: Medico-Legal Implications of Artificial Intelligence-Driven Decision-Making in Orthopedics

**DOI:** 10.3390/bioengineering13020227

**Published:** 2026-02-15

**Authors:** Giuseppe Basile, Vittorio Bolcato, Giulia Bambagiotti, Luca Bianco Prevot, Livio Pietro Tronconi

**Affiliations:** 1 Department of Biomedical Sciences and Public Health, University “Politecnica delle Marche” of Ancona, 60124 Ancona, Italy; 2 Research Center of Legal Medicine and Risk Management, Maria Cecilia Hospital, GVM Care & Research, 48033 Cotignola, Italy; 3 Astolfi Associati Law Firm, 20122 Milan, Italy; 4 Maria Beatrice Hospital, GVM Care & Research, 50121 Florence, Italy; 5 Trauma Unit and Emergency Department, IRCCS Istituto Ortopedico Galeazzi, 20157 Milano, Italy; 6 Department of Health and Life Science, European University of Rome, 00163 Rome, Italy

**Keywords:** artificial intelligence, decision-making support, orthopedics, medico-legal, technology assessment, liability

## Abstract

Orthopedic surgery is undergoing a transformation driven by artificial intelligence (AI), which is reshaping clinico-surgical decision-making. While the operative strategy and professional responsibility traditionally relied on the surgeon’s intuition and manual skills, advanced algorithms now provide predictive, analytical, and procedural decision supports. This paradigm shift is redefining the concept of human error as well as the relationship between technological tools and human decision-makers. As a result, the foundational elements of the healthcare liability framework are being affected. This paper offers a narrative discussion on selected applications of artificial intelligence in orthopedic surgical practice, including patient risk stratification, surgical indication and prosthesis positioning, with a particular focus on the liability implications for healthcare professionals who rely on these systems in terms of therapeutic decision-making. The aim is then to provide a comprehensive medico-legal perspective within the highly regulated and high-risk field of biomedicine, acknowledging and critically assessing the roles and responsibilities of all stakeholders involved—patients, healthcare professionals, innovative technologies, healthcare organizations, and facility management—while balancing innovation, evidence-based practice, and accountability in healthcare delivery.

## 1. Introduction

Orthopedic surgery, like other surgical fields, is characterized by an elevated level of complexity, where the biomechanical, anatomical, and pathological variables of the patient interact dynamically. Achieving a good outcome requires not only the integration of these data but also reliance on the surgeon’s experience, intuition, and relational and manual skills, which play a vital role in decision-making, diagnosis, and fostering informative, empathetic interactions with the user [1,2]. However, complex and repetitive tasks create a performance burden, while navigating large volumes of data hinders effective decision-making and evidence-based choices [3].

From this complexity emerges a concept of professional responsibility understood not merely as technical competence but also as the ability to acknowledge, foresee and control the consequences of one’s actions, to master tools, to learn from experience, including mistakes, lapses and adverse events, and to adjust one’s decision-making process accordingly, and all in a progressive dialogue with the patient [4].

The advent of artificial intelligence (AI) and robot-assisted surgery (RAS) powered by various forms of AI has ushered in a new era in orthopedics. AI is an umbrella term describing various computational technologies capable of processing vast amounts of data, enabling algorithms to learn, interpret, generate predictions, or autonomously decide upon clinical information [5,6]. AI offers the capacity for super-statistical processing of large volumes of data in a coded, systematized, and iterative manner, whose rationale and methods often remain obscure even to its developers, and frequently with a primary purpose that is not healthcare-related [7]. The processing system is therefore rigidly based on the data provided and its quality, reflecting their limitations [8]. The capacity for learning, adaptation, and decision-making is always ultimately rooted in the computational context from which it originates and should not be understood beyond the data or as an ability to differently weigh outcomes based on the broader social impact of errors and responsibilities [9].

The integration into orthopedic surgery of various AI domains, such as machine learning (ML), deep neural networks, large language models (LLMs), video-based and AI-assisted robotics, or augmented reality, has initially shown the ability to support orthopedic care, improve surgical precision, reduce the risk of error and the incidence of adverse events through process standardization, and then optimize clinical outcomes [7,10,11]. Several critical points have been described, including the incomplete controllability and understandability of the methods and outcomes [12], and several hallucinations have been reported in systematic reviews [6,13]. There is also a lack of data weighting and many biases, which are dependent on the quality of the data input and limited internal validation [14].

This issue goes to the core of professional responsibility, which is grounded in the autonomy of healthcare professionals over the decisions they make based on information generated by an increasingly diverse array of tools [15]. It requires examination of several interrelated questions: the extent of the surgeon’s control over AI algorithms, particularly during the sensitive diagnostic phase; the level of decision-making autonomy that surgeons can or should retain in an increasingly AI-driven environment; the problem of algorithmic opacity; and the implications of diverging from algorithmic outputs in relation to established guideline recommendations. Equally relevant are the foreseeability of AI-related adverse events and the evolving role of the surgeon within technology-mediated healthcare processes and organizational structures [5,16].

The integration of surgical expertise with computational power is poised to transform diagnostic pathways and surgical decision-making while simultaneously redefining the framework of professional responsibility [17,18]. These innovations offer substantial potential benefits in terms of effectiveness and efficiency of care through standardization, objective comparability, and data-driven insights [1,19]. Although many studies and systematic reviews have examined the pros and cons of the application of AI in orthopedics [20], few have addressed in a comprehensive manner the medico-legal implications, especially those concerning professional liability in assisted decision-making processes [21,22,23].

### Aim and Methods

Aiming to address this gap in the literature, and considering the intersection with doctrinal juridical debates, we narratively review the medico-legal impact of AI application in orthopedics within the context of European territories. However, what currently emerges is not so much the need to compare the two methodologies underlying therapeutic decision-making but rather the need to initiate a discussion on AI systems so that they can respond not only to the immediate clinical needs of the individual case but also to issues related to healthcare policy and the responsibility of healthcare organizations.

The research was conducted by searching Medline for articles using the keywords “Artificial Intelligence”, “AI AND medico-legal”, “AI AND Orthopedics”, and “AI AND decision-making”. The selected publication period prioritized the last five years (2020–2025). Systematic reviews, meta-analyses, and narrative reviews, published in the last two years (2024–2025), were primarily selected by the first author based on their main impact on orthopedic litigation or on the presence of specific critical medico-legal issues considered worthy of discussion. In the absence of relevant judicial decisions and medico-legal case series related to liability arising from the use of AI in the clinical decision-making process in orthopedics, the approach was exploratory in nature, focusing on the elements underlying responsibility and the identification of causal relationships, necessarily reflecting the authors’ perspectives.

## 2. Medico-Legal Considerations Regarding AI’s Application in Orthopedic Surgery

### 2.1. Diagnostic Phase

The first fundamental aspect that appears in relation to the professional responsibility of the surgical operator is precisely the issue of diagnosis, where AI has offered the most significant and effective solutions, providing improvements in terms of sensitivity and specificity [8], foremost in the radiological diagnosis of various injuries.

Let us consider classic examples from the medico-legal literature on orthopedic malpractice: missed diagnosis and delayed treatment of a fracture in the emergency department based on an X-ray exam and healthcare-associated prosthetic infections [24,25,26].

In the first case, the improvements that AI brings to these diagnostic systems used in the detection of alterations inapparent to human sight or in high-paced settings, however, must be always considered in relation to the professionals who manage the case and their expertise, the availability of such diagnostic tools depending on the healthcare setting, the need to balance sensitivity and specificity in an emergency or elective context, and, of course, economic evaluations of sustainability [27]. Thus, the “consultative” support provided by AI remains distinct from that of a colleague and, ultimately, falls within the physician’s discretionary decision-making, balancing diagnostic capability with a suitable diagnostic–therapeutic pathway [28].

In cases of healthcare-associated infections (HAIs) and surgical site infections, where there is a risk of delayed diagnosis or misdiagnosis, a series of algorithms could be employed sequentially: one to integrate clinical signs and symptoms; another to, based on initial suspicion, recommend specific diagnostic tests and synthesize the results; followed by one that calculates relevant clinical scores; and finally, an algorithm that recommends a targeted therapy tailored to the specific infection and pathogen identified [29,30]. The healthcare providers could limit themselves to reviewing these decision outcomes and going ahead accordingly, with adequate documentation.

Numerous applications have also emerged within the broader field of infection epidemiology, both in hospital and community settings, developed in response to the need for accurate information to support clear decision-making during the COVID-19 pandemic [31]. Applications in tracking infection clusters within hospitals, analyzing microbiological data in the hospital setting, and monitoring patients after discharge for early or late infections, such as for the burden of periprosthetic joint infections, are evident for infection prevention and control policies [32,33]. However, these applications require the structuring and implementation of specific, well-defined algorithms [34].

However, consider precisely the case of suspected periprosthetic infection [35]. AI currently serves as a diagnostic aid, offering a probability-based indication of suspicion. It is then up to the clinician to interpret this information appropriately and start a treatment. Nonetheless, these tools are still limited by the precise collection and integration of data as well as by the availability of second- and third-level diagnostic tests, which depend on the specific clinical setting and established diagnostic–therapeutic pathways, in healthcare facilities of varying organizational and administrative profiles, within poorly digitized and loosely interconnected networks.

This consideration clearly affects the timing and procedures prescribed by institutional protocols and clinical guidelines, ultimately shifting the burden back onto healthcare professionals in the case of potentially preventable harm. In fact, the frequent diagnostic or therapeutic delay related to HAI in orthopedics may find evidence in the presence of a set of AI-generated recommendations, integrated with the health records, which were not adequately or promptly implemented. This would make clear the physician’s non-performance or improper performance in case of litigation [36,37,38]. These two examples of AI applications illustrate possible contrasting scenarios: AI supports the professional, who remains empowered and responsible for decision-making, continuously asked for sensibility and sensitivity selection to avoid over-diagnosis and -treatment. And another scenario where competencies become generalized and there is an over-reliance on AI, which undermines the development of physician clinical skills, attentiveness, and risk assessment abilities [8,39].

An additional risk is the emergence of conflicting or biased outputs, especially if the system is queried multiple times or continuously fed with evolving diagnostic data or low-quality inputs [40].

### 2.2. Preoperative Risk Stratification and Prediction

Concurrently, advanced machine learning models are transforming preoperative risk assessment by enabling more accurate identification of patients at elevated risk of developing complications such as surgical site infections, mechanical implant failures, postoperative pain syndromes, or iatrogenic neurological injuries, sometimes associated with permanent sequelae [41]. In this context, the ability to predict issues such as nerve-related complications or postoperative limb length discrepancies allows for a personalized surgical plan and choice, thereby reducing associated risks and significantly improving long-term outcomes. By analyzing preoperative imaging, such as computed tomography and magnetic resonance imaging, it is possible to generate 3D models of anatomical structures and accurately simulate the surgical procedure [42] or to predict leg length discrepancy in the positioning of prosthetic components in total knee arthroplasty (TKA) [43]. This means that the surgeon is faced with a significantly more complex decision from a medico-legal standpoint.

Indeed, the surgical choice is no longer based on a general assessment of “high” or “low” complication risk but rather on a precise and documented risk score or frequence, such as the case of surgical site infections [44]. This entails taking responsibility for a clearly defined level of risk and implementing specific control measures to reduce the likelihood of its occurrence. For example, this may involve a different preoperative infection screening and decontamination protocol, adjustments to the daily surgical list order, selection of specific antiseptic products, evaluation of postoperative single-bed hospitalization, dressing management strategies, a tighter follow-up schedule, or a tailored informed consent process. In other words, it requires detailed documentation that not only proves the personalization of care but also shows real acknowledgment and management of the specific surgical risk, in terms of both patient information and organizational measures [45,46].

This applies to overall surgical planning and implant design selection in the increasing field of arthroplasty, calling on orthopedic surgeons and overall surgical teams to interpret and finally adapt behaviors according to specific risk indications [47].

### 2.3. Surgical Indication

The issue concerning surgical indication and the informative sources from which it is derived is central and is the main point to be assessed in the malpractice case series: that treatment was adequate for that patient, with those pathological findings and with those coherent clinical guidelines [48,49].

AI, by integrating datasets that are significantly broader than what we are currently accustomed to, and upon which evidence and guidelines are built, can generate scores that figure out the best therapeutic approach for the patient, balancing risks with benefits, then tailoring the care [50]. In other words, it can indicate which category the patient falls into and the level of evidence and/or consensus; for example, between conservative therapy and surgery for lumbar hernia or knee osteoarthritis [51]. And in the case of surgical options, it could indicate which specific approach is most proper, considering minimally invasive options, technology integration and different surgical planning [52].

The benefits and risks assessment must be evaluated according to the best evidence as set in the international and/or national guidelines to offer the patient a care option, and AI support nowadays poorly selects evidence. Later, the surgeon shares the medical decision with the patient, who then chooses their own course of care according to the self-determination principle, based on factors such as quality of life, expectations, work, family, and personal needs that elude computational reasoning [53,54].

Guidelines, however, are abstract references that the surgeon must interpret, considering the specific characteristics and current particular condition of the patient. This, however, may lead to excessive discretion on the part of the individual practitioner in choosing the treatment. Such discretion depends on individual knowledge and experience: a lack of ability, for example, in arthroscopic procedures or the posterior approach in spinal surgery may lead the surgeon to consider alternative options [55,56]. The decision support offered by AI would therefore represent progress in terms of standardization and homogenization of therapeutic choices.

Given the previously mentioned limitations in terms of data quality, the difficulty in accessing a clear, relevant information summary, and issues of inaccuracy or outdated sources, it is evident that, at present, algorithms do not fully meet the need for a transparent, reliable, comparable, and properly referenced information source, one that would protect the surgeon who opts for a specific approach in a specific patient [29].

However, both in the context of transferring health information in individual cases, including for the purpose of the coded completion of hospital discharge summaries, and in the collection of data for research and clinical improvement, certain applications have partially addressed some of the limitations of LLMs [57]. Thus, by confining their use to controlled, domain-specific settings, as within orthopedics, structured algorithms are developed, inputs are standardized and encoded, and benchmarking frameworks are implemented to ensure objective evaluation [58,59]. This approach ultimately enhances performance and strengthens support for the clinical decision-making process.

The role of the surgeon then changes drastically from being aided by a truly structured and autonomous algorithmic decision, which offers the best option in each case. In a process that is documented and supported by AI, the surgeon may nonetheless be required, without completely understanding the algorithm’s reasoning process, to justify any divergence from the surgical recommendation [60,61]. Divergence is, almost by default, to be expected given the current literature limitations, but soon, if the decision-making algorithm is fed with carefully selected data, trained according to precise schemes, peer-reviewed and externally validated, we could assume that any deviation would require even stronger justification [21], especially where there is a streamlining of AI recommendations and their references in a future medical record [62].

The use of predictive algorithms raises a significant issue about informed consent in this phase. Patients must be properly informed not only about the risks associated with the surgical procedure itself but also about the use of technologies that may influence decision-making in selecting indications for surgery [63]. If a physician employs an AI-assisted system for surgical indication and planning but fails to adequately explain the use of such technologies, this may constitute a breach of the duty to inform the patient about potential risks, such as system errors or inaccurate algorithmic decisions, thus limiting, for example, a second opinion before choosing to proceed with the surgery [64].

Even with access to advanced systems offering precise guidance and predictive analytics, surgeons must not lose sight of the fact that their clinical judgment is still paramount. Even with future autonomous clinical decision support systems, the physician keeps ultimate control over the patient’s care and must select the best therapeutic option for the patient.

### 2.4. Intraoperative Phase Decision-Making

Orthopedic robotic surgery employs advanced systems that, through the integration of deep learning algorithms and neural networks, support the surgeon in real-time intra-operative decision-making, both in terms of intraoperative choices and in relation to the generation of documentation, from the surgical report to the comprehensive recording of the surgical team’s activities [11].

Regarding the surgical procedure, however, there is not always a clearly established consensus on the defined steps to be followed during an operation [6]. In this context, an algorithm, prospectively completely autonomous, may not adequately respond to the specific adaptative needs of each case, as it tends to integrate individual surgical processes into a single abstract pathway [65]. New modalities such as augmented reality and video-assisted technologies have the potential to enhance the capabilities of the surgical team and, consequently, improve outcomes [61]. However, they place increased responsibility on professionals to decide how and when to implement these procedures, requiring greater decision-making effort and higher technical competence, translating into a higher standard of diligence [66].

There has also been discussion about using LLM systems to compile medical records and operative notes, such as generating a TKA report based on the typical texts usually written by the surgeon. Even in this context, the professional’s oversight and control are central, since this is not merely about creating a fluent and well-written paragraph but also about providing a reliable basis for clinical information transfer to colleagues and serving as a legal source of evidence. We are already seeing current use of ChatGPT in drafting clinical notes in complex scenarios or summarizing large volumes of prior information into a single entry [67,68]. Once again, these are applications where the AI functions as a tool and responsibility lies with the user, including cases of clear off-label use [69]. It is also true that, considering the documentation gaps that often emerge in medical malpractice litigation, support from these integrated systems, if properly monitored and traceable, could be a step forward in terms of quality and reliability [62,70].

Although documentation needs should not be approached as a burden, they appear complex in multi-professional, multi-level, and highly regulated ecosystems. Nevertheless, it may be beneficial to help with certain documentation and tracking tasks, helping professionals avoid redundant entries and allowing the integration of massive quantities and sources of data (such as blood transfusions, drugs infusions, EMG or MRI recordings, and more).

## 3. Comprehensive Medico-Legal Discussion

### 3.1. Errors and Medico-Legal Perspectives

The presence of predictive algorithms, decision-making algorithms and autonomous robotic systems is indeed reshaping the concept of decision-making in orthopedics, shifting the focus from the individual surgeon’s technical skills to a hybrid paradigm of human–machine interaction. Some of the applications discussed have direct implications not only for clinical practice but also in the medical equipe functioning, procedures adherence, shared responsibility in multidisciplinary settings, and the concept of fallibility in medicine. The consequent need is careful evaluation of technological applications in healthcare while confronting the best clinical evidence and transparency [71].

It is helpful to clarify that an error is defined as the failure of a planned action to be completed as intended, or the use of an incorrect plan to achieve an aim, which may or may not result in harm to the patient [72]. This definition, drawn from the clinical risk management literature, places the error within the broader context of the healthcare system. It views deviation as a systemic outcome, including in the analysis of the role of technology and human–technology interaction, rejecting the notion of individual blame and moving away from focusing solely on the primary actor involved, the healthcare one.

On the other hand, medical malpractice ascertainment typically aims to identify those healthcare behaviors that are inadequate, non-compliant with guideline recommendations or not answering specific patient conditions and known risks [21]. This approach responds to a specific legal question posed by the judicial system and personally shaped: to technically define and attribute the deviation from expected conduct. The latter judicial approach must examine the choices and diagnoses made by professionals in relation to the patient’s pathologies and, in a certain way, tends to overlook the complex external influences on these decisions, an influence that is immensely growing with the use of AI [73].

In a previous model, it was essentially the surgical act itself that was considered decisive, and professional liability was attributed exclusively to the lead surgeon or the surgical team. Today, however, given the high level of technological advancement, the digitalization of surgery, and the horizontal organization of healthcare functions, the issue of liability in healthcare must be approached in a more integrated and systemic manner, like risk management teaches, although the physician remains the grantor of the care and the main player in the specific decision-making process [74].

Future fully autonomous decision-making systems would raise the issue of assigning responsibility to an atypical legal entity, reducing the professional’s ability to exercise control, particularly that of the surgeon [75]. For now, these AI-integrated tools are limited to specific and fragmented tasks, and so other ascribable subjects than physicians seem unrealistic.

The traditional model of responsibility, however, falls short in the context of AI systems, as nobody has enough control over the machine’s actions and functions to be able to assume responsibility for them, as for technology developers [76]. For instance, responsibly for contaminated or broken femoral implant tails will be allocated to the producers, as defects can clearly be traced back to the producers. But how can potential manufacturing defects or errors in development be assessed and attributed to the producers or developers? Can the decision to go ahead despite an unfavorable algorithmic prediction result in professional liability [77]? AI, in fact, is not merely a passive tool but an active system that intervenes in the decision-making process, suggesting operative choices and analyzing data in real time. In this context, the answer to the question of who is liable in case of failure, such as incorrectly predicting a complication, seems straightforward: there is no real concept of error from the AI’s perspective. In fact, it lacks the ability to discern whether an outcome is normal or abnormal and does not possess a value-based understanding of the ultimate purpose of the decision-making process, which remains entrusted to the professional [78].

In an extreme case, the AI-integrated system could suggest turning the placement of a right knee prosthesis because the surgery is being performed improperly on the left knee instead of the right one; we could not definitively say it is making a mistake. It responds to spatial information and thus provides a technically correct answer while being incorrect in the context in which it is applied. Unlike traditional medical devices, it becomes extremely difficult to determine whether the algorithm is malfunctioning or has made a mistake, and thus, to further translate liability to the developers or manufacturers of the system [19].

### 3.2. Legal References and Comparisons in Europe

The current framework, grounded in the paradigmatic principles of civil and criminal liability in the provision of healthcare services, provides certain reference standards for addressing emerging medico-legal issues regarding AI applications in the European and Italian contexts [79,80]. A first point of discussion is certainly that the growing use of generative tools in the surgical field is giving rise to new interpretative vulnerabilities that must be promptly addressed to ensure their proper classification and integration within legal systems.

A broader review of AI deployment has meanwhile been undertaken and incorporated into the EU’s regulatory ecosystem, which is aimed at ensuring patient safety through directly applicable sources, namely Regulation (EU) n. 2024/1689, known as the AI Act (AIA). This regulation is based on a risk-based assessment and preventive control system; however, its full effectiveness remains contingent upon further development and implementation by Member States though the adoption of national provisions [81,82]. The AIA fails to account for the evolving nature of software design and usage: an AI-driven diagnostic tool initially classified and managed as low risk may become high risk as it incorporates real-world patient data. The current version of the AIA is, in fact, a device-type regulation that lacks adaptive oversight mechanisms to address such transitions [77].

Italy has recently enacted a specific regulation with law 23 September 2025, n. 132. The law introduces clear rules for the development, adoption, and use of innovative technologies, mainly addressing use issues. It also establishes the obligation to ensure appropriate training for the professionals required to use them, thereby assigning responsibility to top-level and organizational roles in cases of failure to comply and any resulting harm to individuals [83]. Article 13 addresses the central issue of the role of AI in relation to intellectual professions, like healthcare ones, who exercise discretionary judgment. It clarifies the instrumental value of AI in supporting professionals and the duty to inform patients about AI applications, and thus, clearly affirms their responsibility as final users.

The 2022 UK report and UK Government 2023 White Paper, starting the legislative study on AI use in UK, support the hypothesis of a comprehensive life-cycle product regulatory framework with pre-market risk assessment and post-market surveillance [84,85]. The first document emphasizes the role of healthcare institutions and gatekeepers like the National Institute for Health and Care Excellence (NICE) in facilitating evidence-based updates and the responsible integration and progressive monitoring of these technologies in clinical practice.

Manufacturers currently provide insufficient detail about how model performance varies across different contexts and outside of controlled lab environments, how risks emerge from model validation processes, and how users interact with AI systems. Hence, the medical device regulation (MDR) does not successfully close this gap in ensuring the device’s intended and actual clinical use and does not align with the MDR’s conception of the intended purpose and use, thereby ignoring the societal implications of AI solutions for clinical management [19].

### 3.3. Professionals’ Autonomy

It is essential to recognize that even the most advanced technology of generative AI resolves problems through the repetitive and mechanistic accumulation of data, which are progressively layered and systematized in a continuum, ultimately producing increasingly refined predictive outcomes. Assuming the principle that such technologies serve an auxiliary role in the surgical context, rather than a substitutive one, their integration supports, but does not replace, a clinician’s decisive autonomy [86]. This autonomy is exercised across various layers, including the predictive evaluation of disease progression and treatment outcomes, surgical planning, and the personalization of the surgery. Certainly, AI-assisted systems constitute a valuable enhancement of this model, nourished through the dissemination of data-driven clinical practice and supported by the summarizing efforts of the relevant scientific community. Nevertheless, the healthcare professional is in no way exempt from the level of care and attention required by the management of individual cases. Certainly, the progressive development and tailoring of algorithms will provide a more robust and well-defined foundation for decision-making.

Therefore, in reconstructing clinicians’ conduct, it remains essential to maintain the diagnostic approach to the individual case, an approach that, given the current state of scientific knowledge and the technologies available, cannot be overridden by a wholesale transfer of liability to the AI tools. In the context of forensic expert evaluation, it is thus necessary to consider other potential sources of liability, particularly those relating to the obligations of the manufacturer, the duties of system maintenance upon healthcare management, and the ongoing need to update the information technology systems that allow integrated AI use. This opens up new scenarios in which individuals not belonging to the healthcare professions may be held accountable for their conduct in case of malpractice claims [87].

This broader picture raises important questions about the limitations of the current legal framework and the challenges it poses to interpreters who are called upon to engage with the evolving dynamics of healthcare organization, particularly the functional transformations and the technical–professional hybridization that now inseparably contribute to the provision of surgical care.

## 4. Future Challenges

It will be essential for the scientific community to adopt a multidisciplinary and collaborative approach to address emerging challenges and to optimize the use of these technologies in alignment with evidence-based medicine and medico-legal standards (Table 1).

To this end, we would like to propose recommendations that we consider crucial for guiding the future of AI-assisted orthopedic surgery.

The first step is therefore to define the scope and select the data used to develop and progressively train the algorithm, ensuring the quality and relevance of the input. Certainly, risk and impact assessment mechanisms during the development phase serve as additional filters to promote greater transparency and clinical safety in its eventual application [88,89].

Another critical point concerns the education and training of healthcare professionals in the use of advanced technologies. Despite AI tools increasingly being adopted, both surgeons’ familiarity with them and citizens’ understanding vary considerably, influenced by regional contexts, literacy and resource availability [90]. It is therefore vital, even legally required, to develop specific training programs that include practical simulations and ongoing updates in case of specific applications and single facilities use. Universities and research institutions should actively collaborate with technology companies to ensure that training programs are continuous and of high quality [91].

From a research perspective, there is a growing need for randomized, multicenter trials to evaluate the long-term effectiveness of AI’s application in orthopedic surgery, also addressing the malpractice impact, requiring far longer timing. Such studies should aim to address complex questions regarding the comprehensive implications of these technologies, including improved diagnostic capabilities, optimized surgical decision-making, lower complication rates, and implications for patient safety, ultimately contributing to the broader issue of ensuring clinicians feel secure and supported in the delivery of care [92].

To this end, the scientific community must commit to the development of clear and shared guidelines and/or good clinical practices for managing AI use and the consequent liability [93]. Taking into account the risk scenarios associated with the use of technology, it is essential to clearly define the roles and responsibilities of surgeons, stakeholders, healthcare managers and technology manufacturers to prevent ambiguity that could undermine trust in the profession and place an undue burden on the surgical team [23,94].

The involvement of forensic and legal experts in drafting these guidelines will be crucial. In assessing suitable no-fault legal systems, which can therefore appropriately place the role of healthcare professionals within the broader and more complex context of care and its regulation, it will be necessary to define how to allocate the cost of AI-related damage to society and, in particular, to the AI developers.

## 5. Conclusions

Artificial intelligence represents a revolutionary tool in the field of healthcare, and in orthopedics it offers valuable support in integrating data for clinical decision-making, in comparing and objectifying available evidence, and in preventing complications through enhanced predictive capabilities. However, the full realization of these benefits depends primarily on the optimization, selection, and critical weighting of data sources.

The ability to trace and document the recommendations produced by such tools remains a fundamental prerequisite, especially in support of clinical choices, which, at present, remain the responsibility of the healthcare professional.

Nonetheless, in the case of adverse outcomes associated with AI use, it is essential to accurately determine the level at which the anomaly occurred, considering the entire decision-making chain, involving all stakeholders, from development to commercialization, installation, and ultimately, the clinical use in the individual case by the deployers.

As the final and essentially sole guarantor within the continuum of care, the healthcare professional must be able to rely on robust, evidence-based tools that can support the navigation of increasingly vast and complex bodies of information, particularly in making surgical decisions, such as in orthopedics, so as to engage with the patient in selecting the most appropriate and evidence-based treatment.

## Figures and Tables

**Table 1:** Key points from medico-legal reasoning concerning AI in orthopedics.

Area	Key Point	Summary Description
General Approach	Multidisciplinary and collaborative strategy	Legal and forensic expertise is essential to address emerging challenges and optimize AI use in alignment with evidence-based medicine and medico-legal impact.
Algorithm Development	Advancement of predictive algorithms	Critical for personalizing therapeutic solutions and overall care.
	Data scope and quality definition	Define and select the data used for algorithm development and training, ensuring input quality and relevance.
	Risk and impact assessment mechanisms	Implemented during development to enhance transparency and clinical safety.
Clinical Research	Impact on patient safety and clinician confidence	Studies must also evaluate how AI affects clinician confidence and patients’ safety in care delivery.
Medico-Legal and Regulatory Issues	Clear and shared guidelines and practices	The scientific community must define validated legal frameworks for managing AI-related risks and integrated guidelines.
	Definition of roles and responsibilities	It is crucial to avoid ambiguity on decision-makers and diagnostic responsibilities to protect professional trust and practice.
	Health records and surgical report	Track the decision support system and AI-generated recommendations, include them in informative disclosures to patients, and document the reasons for any deviations.
	Consideration of no-fault legal systems	It should be explored to properly contextualize the shared risks and the role of healthcare professionals within an integrated and technology-driven decision-making process.
	Cost allocation for damages related to AI use	The distribution of costs for such systems must be defined, particularly regarding AI developers.
Professional Training	Targeted education for healthcare professionals	Needed to bridge the skill and literacy gap, which varies by context, role, and experience.
	Interdisciplinary training programs	Should include simulations, continuous updates, and focus on ethical, legal, and social implications of AI, which vary according to persons, resources, and context.
	Institutional collaboration	Universities and research institutions must work with AI developers and tech companies to ensure high-quality, ongoing education.

## Data Availability

Data sharing is not applicable to this article as no new data were created or analyzed in this study.

## Abbreviations

The following abbreviations are used in this manuscript:

AI	Artificial Intelligence
RAS	Robot-Assisted Surgery
HAI	Healthcare-Associated Infection
HDR	Hospital Discharge Record
